# Metabolomics Studies to Assess Biological Functions of Vitamin E Nicotinate

**DOI:** 10.3390/antiox8050127

**Published:** 2019-05-11

**Authors:** Lucia Marcocci, Yuichiro J. Suzuki

**Affiliations:** 1 Department of Biochemical Sciences “A. Rossi Fanelli”, Sapienza University of Rome, 00185 Rome, Italy; Lucia.Marcocci@uniroma1.it; 2 Department of Pharmacology and Physiology, Georgetown University Medical Center, Washington, DC 20057, USA

**Keywords:** cell signaling, tocopherol nicotinate, tocopheryl nicotinate, vitamin E nicotinate

## Abstract

Vitamin E nicotinate (tocopherol nicotinate, tocopheryl nicotinate; TN) is an ester of two vitamins, tocopherol (vitamin E) and niacin (vitamin B3), in which niacin is linked to the hydroxyl group of active vitamin E. This vitamin E ester can be chemically synthesized and is used for supplementation. However, whether TN is formed in the biological system was unclear. Our laboratory previously detected TN in rat heart tissues, and its level was 30-fold lower in a failing heart (Wang et al., *PLoS ONE* **2017**, *12*, e0176887). The rat diet used in these experiments contained vitamin E acetate (tocopherol acetate; TA) and niacin separately, but not in the form of TN. Since only TN, but not other forms of vitamin E, was decreased in heart failure, the TN structure may elicit biologic functions independent of serving as a source of active vitamin E antioxidant. To test this hypothesis, the present study performed metabolomics to compare effects of TN on cultured cells to those of TA plus niacin added separately (TA + N). Human vascular smooth muscle cells were treated with TN or with TA + N (100 μM) for 10 min. Metabolite profiles showed that TN and TA + N influenced the cells differentially. TN effectively upregulated various primary fatty acid amides including arachidonoylethanoamine (anandamide/virodhamine) and palmitamide. TN also activated mitogen-activated protein kinases. These results suggest a new biological function of TN to elicit cell signaling.

## 1. Introduction

Vitamin E nicotinate (tocopherol nicotinate, tocopheryl nicotinate; TN) is an ester of two vitamins: tocopherol (vitamin E) and niacin (vitamin B3). Vitamin E exerts potent antioxidant activities through its hydroxyl group of the chromanol ring. In the TN structure, this hydroxyl group is esterified to link niacin. TN can be chemically synthesized and is commercially available for supplementation [1]. However, its use has been overshadowed by another more popular vitamin E ester supplement, vitamin E acetate (tocopheryl acetate; TA), in which acetate is bound to the hydroxyl group of vitamin E. Thus, published literature concerning TN is limited, and the biology of TN is not well understood [1]. Although the TN structure can be derived by adding two essential vitamins that are incorporated into the body through diet, supplementation or both, whether the TN molecule can be formed in the biological system is not known.

Recently, metabolomics studies performed in our laboratory revealed that rat heart tissues contain TN [2]. The rat diet used in these experiments only contained TA and niacin separately, but not in the form of TN. Therefore, the biological system appears to possess a mechanism to generate TN, likely from niacin and vitamin E. Moreover, the heart tissue level of TN was decreased 30-fold when rats were subjected to the pulmonary hypertension-induced right heart failure, providing the possible clinical importance of endogenous TN [2]. Although these failing hearts are expected to be subjected to oxidative stress and thus decrease the endogenous levels of various active antioxidants, our experiments did not show other forms of vitamin E being decreased in heart failure. Thus, we propose that the TN structure per se confers biologic activities independent of serving as a source of active vitamin E and acting as an antioxidant [1,2].

To further test this concept, we treated cultured human cells with either TN or TA plus niacin added separately (TA + N) and compared changes in the levels of cellular small molecules by metabolomics analysis. These results as well as our analysis of protein kinase activation suggest that TN elicits cell signaling, providing a new biological role of this molecule.

## 2. Materials and Methods

### 2.1. Chemicals

DL-alpha-TN (Catalog # T5134), DL-alpha-TA (Catalog # T3376), and niacin (Catalog # PHR1276) were purchased from Sigma-Aldrich (St. Louis, MO, USA). TN and TA were dissolved in ethanol and niacin was dissolved in water to make 100 mM stock solutions.

### 2.2. Cell Culture

Human pulmonary artery smooth muscle cells purchased from ScienCell Research Laboratories (Carlsbad, CA, USA) were cultured in accordance with the manufacturer’s instructions in 5% CO_2_ at 37 °C. To reduce basal cell signaling events, cells were serum-starved overnight with smooth muscle cell growth medium (ScienCell) supplemented with 0.4% fetal bovine serum and 1% penicillin/streptomycin. Cells were treated with TN (final concentration, 100 µM), with a combination of TA (100 µM) plus niacin (100 µM) (TA + N) or with niacin (100 µM). The final concentration of ethanol that was used as a vehicle for TN and TA was 0.1% (*v/v*).

### 2.3. Metabolomics

After treatment with TN or TA + N for 10 min, cells (1 × 10^7^ cells per sample) were washed in phosphate buffered saline (PBS) three times and collected by scraping. Cells were then centrifuged at 1500 rpm at 4 °C for 10 min and stored at −80 °C. For extraction, cell pellets were resuspended in water, placed in dry ice for 30 s, followed by incubation at 37 °C for 90 s. This freeze-thaw cycle was repeated, and samples were sonicated for 30 s. Methanol containing internal standards was added, vortexed, and incubated on ice for 15 min. This was followed by the addition of chloroform at room temperature, vortexing, and centrifugation at 13,000 rpm for 10 min. Chilled acetonitrile was added to each sample tube, and the samples were vortexed and incubated overnight at −20°C. Samples were then centrifuged at 13,000 rpm for 10 min at room temperature, and the supernatants transferred to fresh tubes and dried under vacuum. The dried metabolite mixtures were resuspended in 100 μL of 50% methanol for mass spectrometry analysis. 

Each sample (5 μL) was injected onto a reverse-phase 50 × 2.1 mm ACQUITY 1.7-μm C18 column on an ACQUITY ultra-performance liquid chromatography (UPLC) system (Waters Corporation, Milford, MA, USA) with a gradient mobile phase consisting of 2% acetonitrile in water containing 0.1% formic acid and 2% water in acetonitrile containing 0.1% formic acid. Each sample was resolved for 10 min at a flow rate of 0.5 mL/min. The gradient started with 95% A and 5% B for 0.5 min with a ramp curve. At 8 min, the gradient reached 2% A and 98% B. From 8 to 9 min, the gradient shifted to 0% A and 100% B and shifted back to 95% A and 5% B from 9 to 10 min. 

The column eluent was introduced directly into the mass spectrometer by electrospray. Mass spectrometry was performed on a Quadrupole-time-of-flight (Q-TOF) Premier (Waters) operating in either positive-ion (ESI+) or negative-ion (ESI−) electrospray ionization mode with a capillary voltage of 3200 V and a sampling cone voltage of 20 V in negative mode and 35 V in positive mode. Data were acquired in centroid mode from 50 to 850 *m/z* in mass spectrometry scanning. Peak detection was performed using XCMS software (Scripps Research Institute, San Diego, CA, USA). Total ion chromatograms are shown in the Figure S1.

### 2.4. Western Blotting

Equal protein amounts of cell lysate samples were electrophoresed through reducing sodium dodecyl sulfate (SDS) polyacrylamide gels and electroblotting onto nitrocellulose membranes (Bio-Rad Laboratories, Hercules, CA, USA) [3]. Membranes were then blocked with 5% bovine serum albumin and incubated with primary antibodies for phospho-ERK1/2 (Thr202/Tyr204; Catalog # 4370; Cell Signaling Technology, Danvers, MA, USA) or ERK1/2 protein (Catalog # 9102; Cell Signaling). Membranes were washed and incubated with horseradish peroxidase-linked secondary antibodies, and Enhanced Chemiluminescence (ECL) System (GE Healthcare Bio-Sciences, Pittsburgh, PA, USA) was used for detection. Autoradiography was performed using UltraCruz Autoradiography Films (Santa Cruz Biotechnology, Dallas, TX, USA), and optical densities of protein bands were quantified using ImageJ (National Institutes of Health, Bethesda, MD, USA).

### 2.5. Statistical Analysis

Means and standard errors were computed. Statistical comparisons between two groups were performed by two-tailed Student’s *t-*test, and three or more groups were analyzed by using one-way analysis of variance (ANOVA). Differences at *p* < 0.05 were defined as significant.

## 3. Results

**Figure 1:**
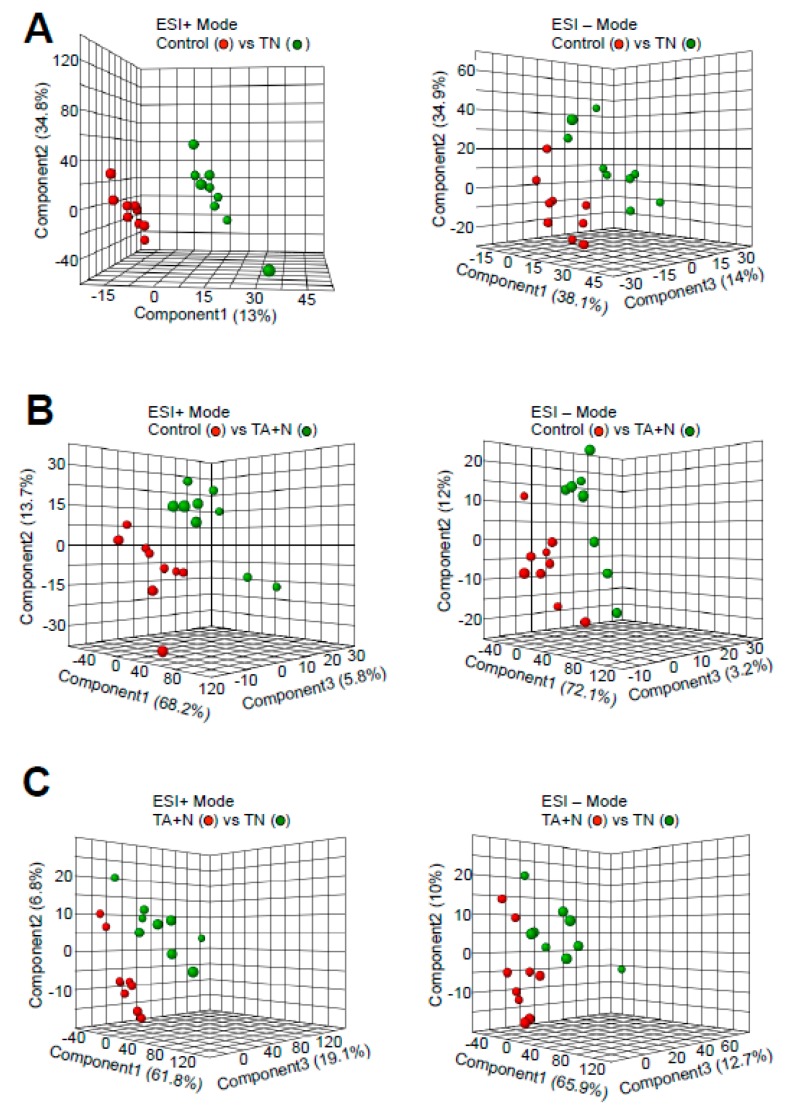
Partial least-squares discriminant analysis (PLS-DA) plots. (**A**) ESI+ and − modes of control vs. vitamin E nicotinate (TN); (**B**) ESI+ and − modes of control vs. vitamin E acetate + niacin (TA + N); (**C**) ESI+ and − modes of TN vs. TA + N. Human pulmonary artery smooth muscle cells were treated with either TN (100 µM) or with TA (100 µM) plus niacin (100 µM) (TA + N) for 10 min. Samples were subjected to metabolomics analysis (*n* = 9). ESI: electrospray ionization.

Partial least-squares discriminant analysis (PLS-DA) plots derived from metabolomics analysis showed that metabolomics profiles (red circles vs. green circles) were clearly different when cells were treated with either TN (Figure 1A) or TA + N (Figure 1B) compared to an untreated control, suggesting that both treatments altered cellular metabolite contents. Figure 1C also shows that the profiles between TN and TA + N treatments were also clearly different, indicating that TN exerts different effects on the cells compared to vitamin E and niacin added separately. 

**Figure 2:**
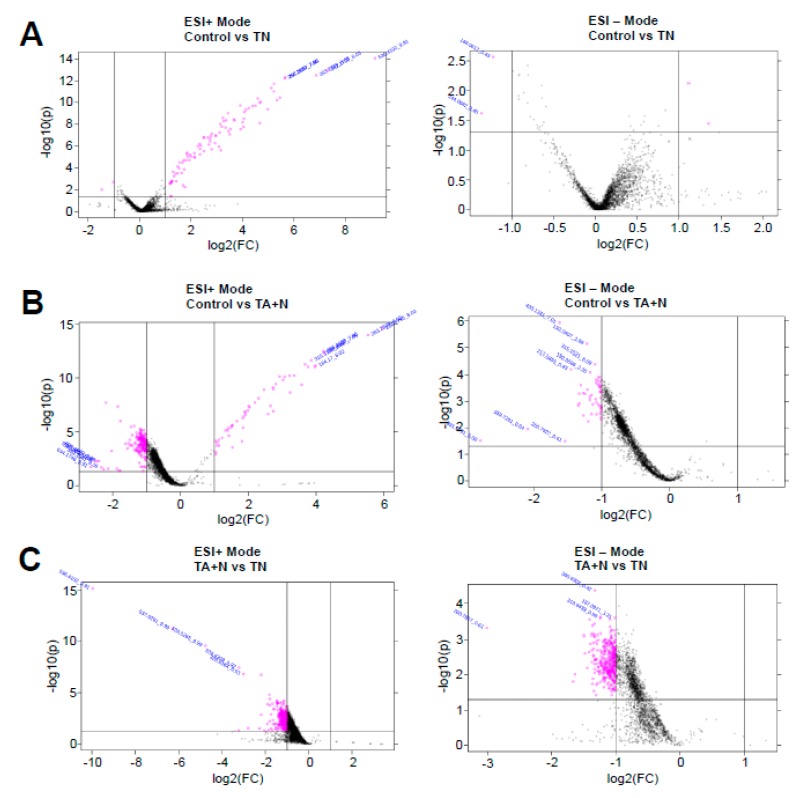
Volcano plots. (**A**) ESI+ and − modes of control vs vitamin E nicotinate (TN); (**B**) ESI+ and − modes of control vs. vitamin E acetate + niacin (TA + N); (**C**) ESI+ and − modes of TN vs. TA + N. Human pulmonary artery smooth muscle cells were treated with either TN (100 µM) or with TA (100 µM) plus niacin (100 µM) (TA + N) for 10 min. Samples were subjected to metabolomics analysis (*n* = 9). The dot plots represent fold changes (FC) on the x-axis and statistical significance is shown by the *p*-value (p) on the y-axis. Pink dots represent values with *p* ≤ 0.05 and a multiplicative change of greater than two-fold.

The pink dots shown in Figure 2 indicate molecules that were found to be differentially expressed with at least 2-fold difference with *p* < 0.05 for both ESI+ and ESI− modes in the mass spectrometry analysis. Since we are specifically interested in the differences between the effects of TN and TA + N, the metabolomics profiles of pink dots shown in Figure 2C that depict small molecules that are differentially expressed between TN and TA + N groups were further analyzed.

Metabolomics identified a total of 2828 molecules with a molecular mass of ≤1200 to be differentially expressed between the TN and TA + N groups (1643 in positive mode and 1185 in negative mode). Many of the molecules with an identified experimental mass did not correspond to currently known molecules. Some experimental masses corresponded with synthetic chemicals that are not expected to occur in our human cell culture system; thus, these are likely to be unknown molecules as well. Detailed examinations of the list of metabolites revealed that many molecular masses identified corresponded to biologically relevant lipid molecules (Supplemental File S1), and fewer were found to be small peptides (Supplemental File S2). 

In Supplemental File S1, one notable lipid molecule that was found to be differentially expressed between TN and TA + N groups was, not surprisingly, the TN molecule. TN was found to be >1000-fold higher in TN-treated cells compared to TA + N-treated cells, as TN was added to cultured cells. These results also indicate that adding TA and niacin separately to human vascular smooth muscle cells does not form appreciable levels of the TN molecule, at least not within 10 min.

**Figure 3:**
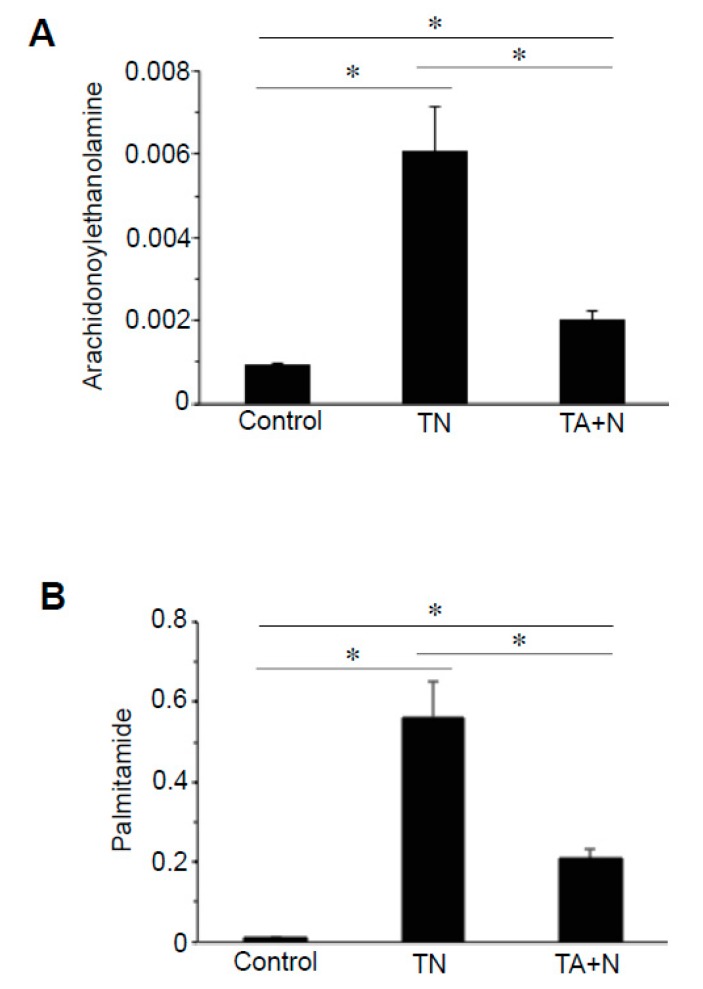
TN is an efficient activator of arachidonoylethanolamine and palmitamide upregulation. Human pulmonary artery smooth muscle cells were treated with either TN (100 µM) or TA (100 µM) plus niacin (100 µM) (TA + N) for 10 min. Samples were subjected to metabolomics analysis. (**A**) Arachidonoylethanolamine and (**B**) palmitamide were found to be differentially expressed between TN and TA + N groups. Bar graphs represent the mean ± SEM values of signal intensity. * Values are significantly different from each other at *p* < 0.05 (*n* = 9).

Treatment with TN also affected the cellular expression of derivatives of various fatty acids, particularly *N*-acetylethanolmines and primary fatty acid amides. Arachidonylethanolamine is a notable molecule that can be either anandamide or virodhamine, which have the same mass, given the same carbon chain length. Arachidonylethanolamines of different lengths were found to be higher in TN-treated cells compared with TA + N-treated cells. Figure 3A shows that the reason for the differential expression of arachidonylethanolamines between TN and TA + N groups is because the TN molecule was three times more efficient in increasing arachidonylethanolamines than TA + N. These results suggest that TN is capable of eliciting biologic responses independent of vitamin E or niacin. Differential expression of primary fatty acid amides such as oleamide, elaidamide, stearamide, linoleamide, palmitoleamide, and palmitamide (Figure 3B) were also observed in TN vs. TA + N cells. 

**Figure 4:**
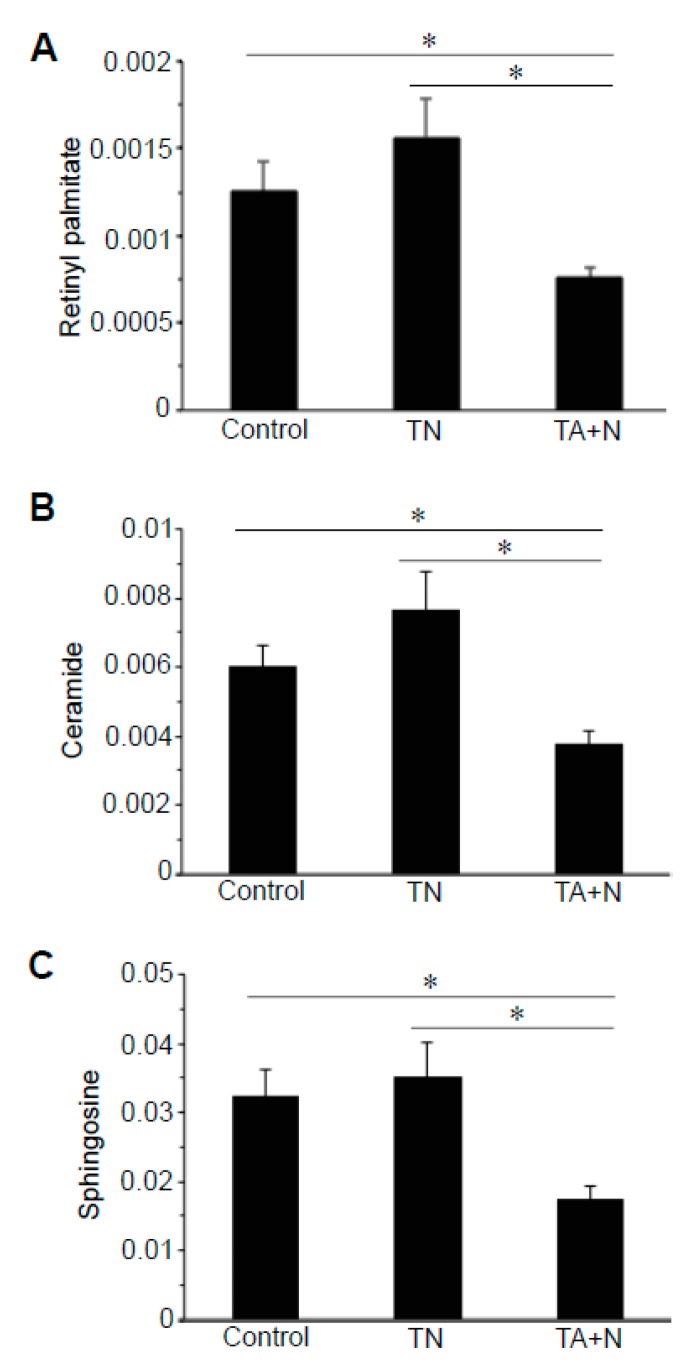
TA + N, but not TN, downregulates retinyl palmitate, ceramide, and sphingosine. Human pulmonary artery smooth muscle cells were treated with either TN (100 µM) or TA (100 µM) plus niacin (100 µM) (TA + N) for 10 min. Samples were subjected to metabolomics analysis. (**A**) Retinyl palmitate, (**B**) ceramide, and (**C**) sphingosine were found to be differentially expressed between TN and TA + N groups. Bar graphs represent mean ± standard error of the mean (SEM) values of signal intensity. * Values are significantly different from each other at *p* < 0.05 (*n* = 9).

**Figure 5:**
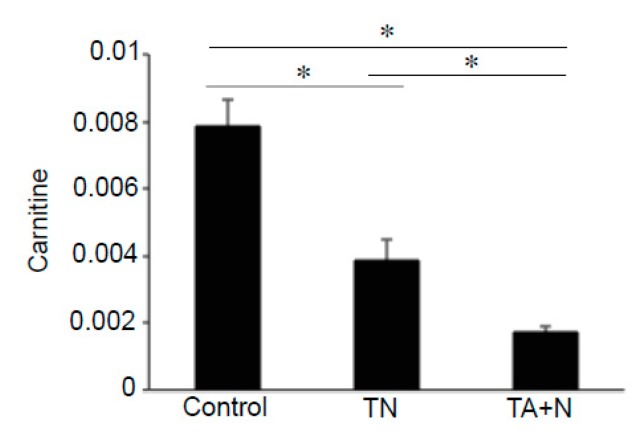
Effects of TN and TA + N on carnitine. Human pulmonary artery smooth muscle cells were treated with either TN (100 µM) or TA (100 µM) plus niacin (100 µM) (TA + N) for 10 min. Samples were subjected to metabolomics analysis. Carnitine was found to be differentially expressed between TN and TA + N groups. The bar graphs represent mean ± SEM values of signal intensity. * Values are significantly different from each other at *p* < 0.05 (*n* = 9).

By contrast, Figure 4 shows that molecules including retinyl palmitate, ceramide, and sphingosine exhibited differential responses to TN and TA + N because TA + N, but not TN, downregulated the levels of these molecules. Figure 5 shows that both TN and TA + N significantly downregulated carnitine, but TA + N was more effective in doing so than TN.

**Figure 6:**
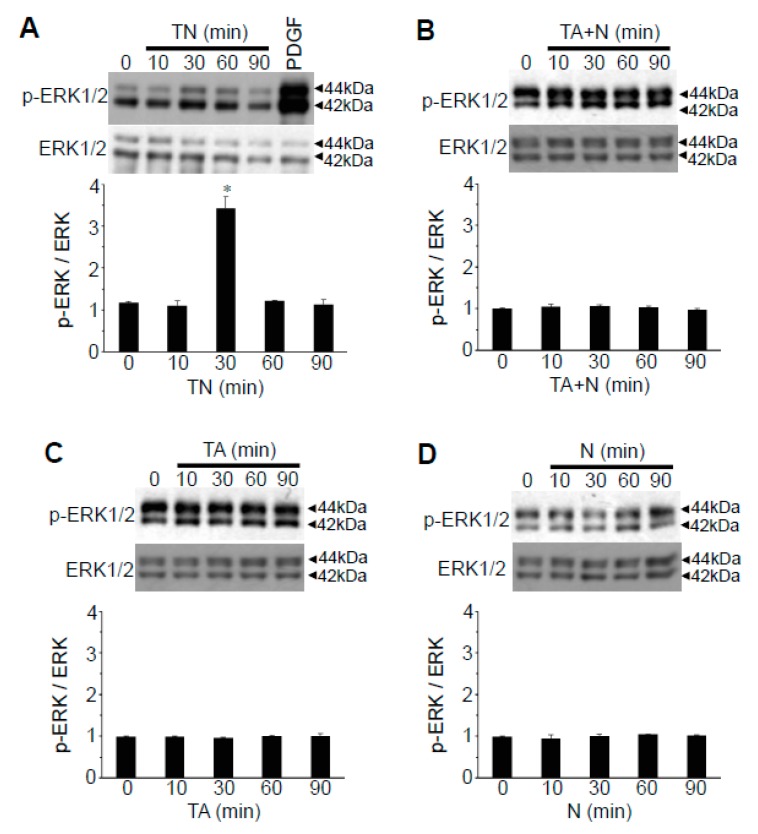
TN activates extracellular signal regulated kinase (ERK) mitogen-activated protein (MAP) kinase. (**A**) Human pulmonary artery smooth muscle cells were treated with TN (100 µM) for the durations indicated or with platelet-derived growth factor (PDGF; 10 ng/mL) for 10 min as a positive control. Cell lysates were subjected to Western blotting for phosphorylated ERK1/2 (p-ERK) and ERK1/2 protein. The bar graph represents the means +/− SEM of the p-ERK/ERK ratio. * Significantly different from control at *p* < 0.05 (*n* = 4). (**B**–**D**) Cells were treated with either TA (100 µM) plus niacin (100 µM; TA + N), TA (100 µM), or niacin (100 µM; N) for the durations indicated. Cell lysates were subjected to Western blotting for phosphorylated ERK (p-ERK) and ERK1/2 protein. The bar graph represents the means +/− SEM of p-ERK/ERK ratio. Values are not significantly different.

Since the results in Figure 3 imply that the intact TN structure may serve as a cell signaling activator, we tested whether TN can activate extracellular signal regulated kinase (ERK) mitogen-activated protein (MAP) kinase, the major protein kinase that regulates cell signaling. While the effects were mild, we found significant increases in p44 and p42 ERK MAP kinase phosphorylation levels in response to TN treatment of cultured human vascular smooth muscle cells with a peak at 30 min (Figure 6A). Neither TA + N added separately (Figure 6B), TA alone (Figure 6C), nor niacin alone (Figure 6D) activated ERK MAP kinases.

## 4. Discussion

We previously reported that TN is expressed in the heart, and its level is dramatically reduced in heart failure in a rat model [2]. Such reduction was only found for TN but not for any other vitamin E molecules, and the rat diet did not contain TN, but contained TA and niacin separately [2]. From these results, we hypothesized that TN can be formed in biological systems and that TN may function not only through the formation of vitamin E or niacin, but while maintaining the TN structure [1,2]. In the present study, the hypothesis that TN can elicit biological activities that are distinct from known functions of vitamin E and niacin was tested by performing metabolomics profiling analysis of cell samples that were treated with either TN or TA + N.

The results shown in Figure 1C indicate that the TN group and the TA + N group were clearly separated, suggesting that these two treatments elicit differential biological responses. Metabolomics profiling identified a number of small molecules that were differentially affected by the two treatments, as indicated as pink dots in Figure 2C. Since these metabolomics data are defined by the masses of molecules, one given mass often corresponds to multiple molecules. Thus, in many cases, it was not possible to pinpoint biologically relevant molecules that were differentially affected by the two treatments. However, through the detailed analysis of a number of molecules, we determined with high confidence that arachidonoylethanolamine, palmitamide, retinyl palmitate, ceramide, sphingosine, and carnitine were differentially influenced by TN and TA + N. Among them, arachidonoylethanolamine and palmitamide were found to be selectively increased by TN.

*N*-acylethanolamines, including anandamide (*N*-arachidonoylethanolamine) and virodhamine (*O*-arachidonoylethanolamine), and primary fatty acid amides, including palmitamide, belong to the long-chain fatty acid amides family of bioactive lipids. These molecules have been identified in the brain as well as in the peripheral tissues, and they modulate various physiological functions [4,5]. Anandamide is an agonist for the endocannabinoid CD1 and CD2 receptors [6] and also reacts with peroxisome proliferator-activated receptor-α (PPAR-α), TPRV1 and TRPM8 receptor channels [7,8,9]. Virodhamine is an agonist of CB1 and CB2 receptors [10] and also acts on the orphan receptor GRP55 [11]. Primary fatty acid amides including palmitamide have also been demonstrated to be important cell signaling molecules [12]. Palmitamide activates PPARα and upregulates the synaptic function of hippocampal mouse neurons [13].

Various enzymes can regulate the synthesis and catabolism of *N-*acylethanolamine. A Ca^2+^-dependent transacylase can transfer the sn-1 acyl chain of phospholipids onto the primary amine of phosphatidylethanolamine to generate *N*-acyl phosphatidylethanolamine, which might be hydrolyzed by phospholipase D to produce *N*-acylethanolamines and phosphatidic acid [14]. *N*-acylethanolamines can be also formed through the catalysis by phosphatase, α,β-hydrolase 4, lysophospholipase D, phosphodiesterase, phospholipase A_2_, phospholipase C, and phosphatase [15,16]. Thus, these pathways may be selectively activated by TN. While our results do not provide information about whether TN-mediated formations of arachidonoylethanolamine and palmitamide are in the same pathway, *N*-acylethanolamines might be the precursors for palmitamide [17,18]. As an alternative to the activation of the formation of arachidonoylethanolamine and palmitamide, TN may inhibit the catabolism of these molecules. *N*-acylethanolamines can be catabolized by fatty acid amide hydrolases [19] as well as by lysosomal acid amidase [20]. Primary fatty acid amides are hydrolyzed by fatty acid amide hydrolase [21,22]. Thus, TN signaling may inhibit these enzymes.

We propose that TN elicits cell signaling through the receptor-mediated process to activate these synthetic pathways or to inhibit the catabolic pathways for increasing *N*-acylethanolamines and fatty acid amides. Further work is needed to identify the TN receptors; however, it should be noted that niacin has been shown to bind to G-protein coupled receptor 109A (GPR109A) [23]. Since TN is more efficient in increasing fatty acid amides, TN may bind to this receptor even more efficiently than niacin.

We also found that TN, but not niacin, TA or TA + N, activates ERK MAP kinase. While our results have not established a causal relationship, TN may activate the MAP kinase pathways through fatty acid amides, taking into consideration that stearoyl ethanolamide has been reported to activate the ERK MAP kinase pathway [5] and anandamide is an activator of p38-MAPK signaling [24].

## 5. Conclusions

In summary, the present study substantiated our hypothesis that TN elicits cellular responses independent of merely serving as a source of vitamin E or niacin. These results along with our previous studies [1,2] collectively suggest an exciting idea of the new biological role for TN.

## Supplementary Materials

The following are available online at https://www.mdpi.com/2076-3921/8/5/127/s1, Figure S1: Total ion chromatograms, File S1: List of masses identified to be differentially affected by TN vs. TA + N that correspond to lipids, File S2: List of masses identified to be differentially affected by TN vs. TA + N that correspond to peptides.
