# Supplementary material for: Metabolomics Studies to Assess Biological Functions of Vitamin E Nicotinate
Source: Antioxidants (Basel). 2019 May 11;8(5):127. doi: 10.3390/antiox8050127 (PMC6562962; doi:10.3390/antiox8050127)
Supplement: Supplementary file 1 [file antioxidants-08-00127-s001.pdf]

Supplemental Figure

Metabolomics studies to assess biological functions of vitamin E nicotinate

Lucia Marcocci[a] and Yuichiro J. Suzuki[b]

[a]Department of Biochemical Sciences "A. Rossi Fanelli", Sapienza University of Rome, 00185 Rome, Italy

[b]Department of Pharmacology and Physiology, Georgetown University Medical Center, Washington, DC 20057 USA

Figure S1

Total ion chromatogram for ESI+

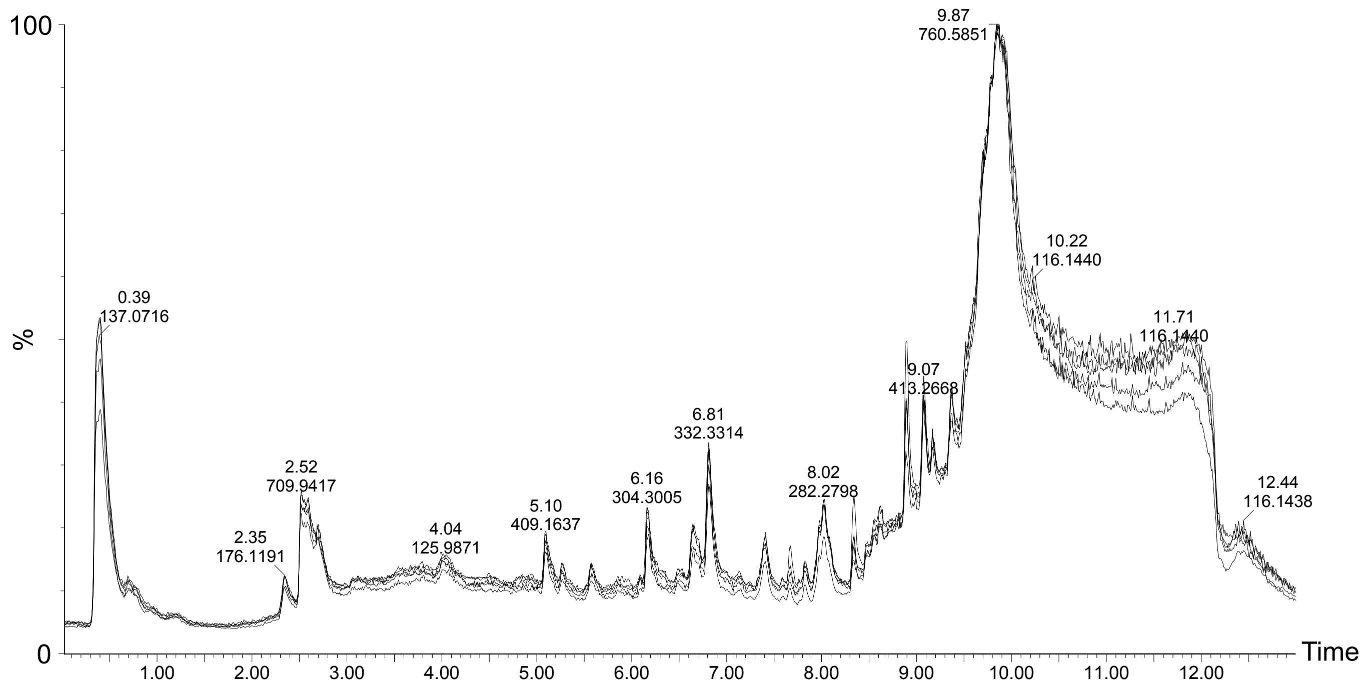

Total ion chromatogram for ESI-

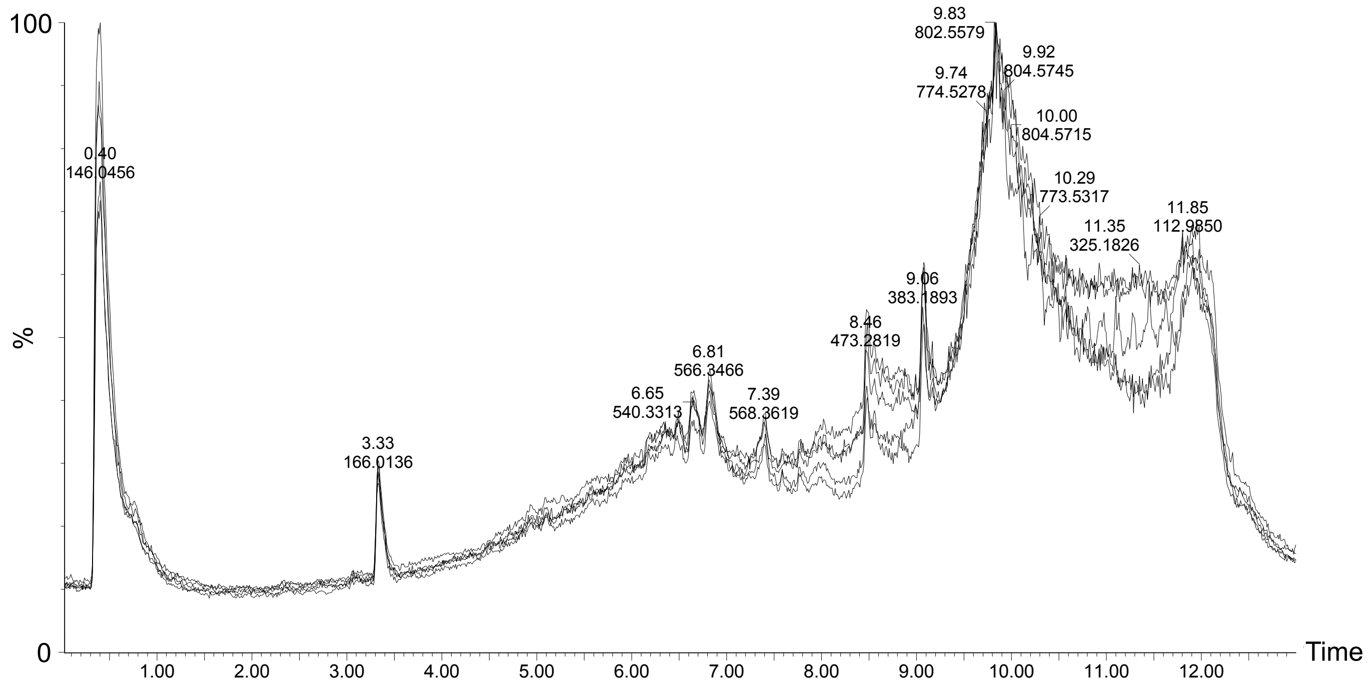

Supplemental Files

Metabolomics studies to assess biological functions of vitamin E nicotinate

Lucia Marcocci[a] and Yuichiro J. Suzuki[b]

[a]Department of Biochemical Sciences "A. Rossi Fanelli", Sapienza University of Rome, 00185 Rome, Italy

[b]Department of Pharmacology and Physiology, Georgetown University Medical Center, Washington, DC 20057 USA

Supplemental File S1: List of masses identified to be differentially affected by TN vs. TA+N that correspond to lipids

Positive mode

Experimental mass: 536.4102
Fold change (TA+N / TN): 0.0010047
P value: 0.00000000000000749
    dl-alpha-Tocopherol nicotinate

Experimental mass: 354.3348
Fold change (TA+N / TN): 0.2839
P value: 0.0000152
    Anandamide (20:l, n-9)

Experimental mass: 376.3189
Fold change (TA+N / TN): 0.28947
P value: 0.0000396
    N-(5Z,8Z,11Z,14Z-docosatetraenoyl)-ethanolamine
    Adrenoyl-EA
    alpha,alpha-dimethyl anandamide
    N-(5Z,8Z,11Z,14Z-docosatetraenoyl)-ethanolamine
    N-(1,1-dimethyl-2-hydroxy-ethyl) arachidonoyl amine
    N-ethyl N-(2-hydroxy-ethyl) arachidonoyl amine
    Anandamide (20:l, n-9)

Experimental mass: 256.2639
Fold change (TA+N / TN): 0.37363
P value: 0.00010254
    Palmitamide

Experimental mass: 378.331
Fold change (TA+N / TN): 0.31426
P value: 0.00011665
    Eicosanoyl-EA
    Guazatine

Experimental mass: 320.2558
Fold change (TA+N / TN): 0.27002
P value: 0.00012562
    Anandamide (18:4, n-3)
    N-hydroxy arachidonoyl amine
    (Z)-N-(2-hydroxyethyl)hexadec-7-enamide
    Cassine
    Spiroxamine
    3-ketosphingosine

Palmitoleoyl-EA
2S-amino-octadeca-4E,6E-diene-1,3R-diol
3-ketosphingosine
4E,14Z-Sphingadiene
(4E,8E,d18:2) sphingosine
(4E,8Z,d18:2) sphingosine
Lepadin D

Experimental mass: 348.2867
Fold change (TA+N / TN): 0.33043
P value: 0.00036519
O-Arachidonoyl Ethanolamine
N-(7Z,10Z,13Z,16Z-docosatetraenoyl)-ethanolamide
Anandamide (20:4, n-6)
Virodhamine
N-oleoyl ethanolamine

Experimental mass: 310.3108
Fold change (TA+N / TN): 0.41919
P value: 0.00053591
Oleoyl Ethyl Amide
N-Hexadecanoylpyrrolidine

Experimental mass: 460.2464
Fold change (TA+N / TN): 0.42446
P value: 0.00080586
Cerivastatin
13-Desoxyterpendole I
beta-Paxitriol
PC(O-10:1(9E)/2:0)
PE(15:1(9Z)/0:0)

Experimental mass: 282.2795
Fold change (TA+N / TN): 0.39156
P value: 0.0011243
Dodemorph
Oleamide
Elaidamide

Experimental mass: 752.5217
Fold change (TA+N / TN): 0.49992
P value: 0.0011482
PE(18:4(6Z,9Z,12Z,15Z)/19:1(9Z))
PE(18:4(6Z,9Z,12Z,15Z)/19:1(9Z))
PE(19:1(9Z)/18:4(6Z,9Z,12Z,15Z))
(Partial listing)

Experimental mass: 322.2717
Fold change (TA+N / TN): 0.30181
P value: 0.0012571
Anandamide (18:3, n-3)
Anandamide (18:3, n-6)
L-threo-Sphingosine C-18
2R-aminooctadec-4Z-ene-1,3S-diol
Sphingosine
Palmitoyl-EA
3-ketosphinganine
Sphingosine
12-amino-octadecanoic acid
2-amino-octadecanoic acid
Sphingosine
5-hydroxy,3E-sphingosine
(8Z,d18:1) sphingosine

Experimental mass: 421.3126
Fold change (TA+N / TN): 0.44451
P value: 0.0013067
1alpha-hydroxy-22-(3-methylphenyl)-23,24,25,26,27-pentanorvitamin D3

Experimental mass: 525.4709
Fold change (TA+N / TN): 0.4845
P value: 0.0014075
all-trans-retinyl Palmitate
11-cis-retinyl palmitate
Linolenyl linolenate

Experimental mass: 426.3538
Fold change (TA+N / TN): 0.44338
P value: 0.0023548
Elaidic carnitine
O-oleoylcarnitine
11Z-Octadecenylcarnitine
Oleoylcarnitine
Octadecenoylcarnitine
Vaccenyl carnitine

Experimental mass: 545.397
Fold change (TA+N / TN): 0.44229
P value: 0.0025414
Panaxydol linoleate

Experimental mass: 619.4357
Fold change (TA+N / TN): 0.49666
P value: 0.0032261

- 3,6-Epoxy-5,5',6,6'-tetrahydro-b,b-carotene-3',5,5',6'-tetrol
- PA(17:1(9Z)/13:0)
- PA(18:1(9Z)/12:0)
- PA(13:0/17:1(9Z))
- PA(14:1(9Z)/16:0)
- PA(15:0/15:1(9Z))
- PA(15:1(9Z)/15:0)
- PA(14:0/16:1(9Z))
- PA(12:0/18:1(9Z))
- PA(16:0/14:1(9Z))
- PA(16:1(9Z)/14:0)

Experimental mass: 843.524
Fold change (TA+N / TN): 0.45725
P value: 0.003386

- Spiramycin 1
- PG(22:6(4Z,7Z,10Z,13Z,16Z,19Z)/20:4(5Z,8Z,11Z,14Z))
- PG(20:4(5Z,8Z,11Z,14Z)/22:6(4Z,7Z,10Z,13Z,16Z,19Z))

Experimental mass: 608.602
Fold change (TA+N / TN): 0.39738
P value: 0.0037891

- Cer(d16:1/23:0)
- Cer(d15:1/24:0)

Experimental mass: 650.4363
Fold change (TA+N / TN): 0.48641
P value: 0.0040958

- PC(16:0/9:0(CHO))

Experimental mass: 254.2472
Fold change (TA+N / TN): 0.40412
P value: 0.0049162

- Palmitoleamide

Experimental mass: 819.5243
Fold change (TA+N / TN): 0.48035
P value: 0.0050407

- PG(22:6(4Z,7Z,10Z,13Z,16Z,19Z)/18:2(9Z,12Z))
- PG(20:4(5Z,8Z,11Z,14Z)/20:4(5Z,8Z,11Z,14Z))
- PG(20:3(8Z,11Z,14Z)/20:5(5Z,8Z,11Z,14Z,17Z))
- PG(20:5(5Z,8Z,11Z,14Z,17Z)/20:3(8Z,11Z,14Z))
- LBPA(20:4(5Z,8Z,11Z,14Z)/20:4(5Z,8Z,11Z,14Z))

PG(18:4(6Z,9Z,12Z,15Z)/22:4(7Z,10Z,13Z,16Z))
PG(22:4(7Z,10Z,13Z,16Z)/18:4(6Z,9Z,12Z,15Z))
PG(18:3(6Z,9Z,12Z)/22:5(7Z,10Z,13Z,16Z,19Z))
PG(18:3(9Z,12Z,15Z)/22:5(7Z,10Z,13Z,16Z,19Z))
PG(18:3(9Z,12Z,15Z)/22:5(4Z,7Z,10Z,13Z,16Z))
PG(18:3(6Z,9Z,12Z)/22:5(4Z,7Z,10Z,13Z,16Z))
PG(18:2(9Z,12Z)/22:6(4Z,7Z,10Z,13Z,16Z,19Z))

Experimental mass: 829.5704
Fold change (TA+N / TN): 0.48177
P value: 0.0052563
PA(22:1(11Z)/22:4(7Z,10Z,13Z,16Z))
PA(22:4(7Z,10Z,13Z,16Z)/22:1(11Z))

Experimental mass: 356.2745
Fold change (TA+N / TN): 0.46424
P value: 0.0055133
6,9,12,15,18,21-Tetracosahexaenoic acid

Experimental mass: 300.2901
Fold change (TA+N / TN): 0.49945
P value: 0.006185
L-threo-Sphingosine C-18
2R-aminooctadec-4Z-ene-1,3S-diol
Sphingosine
Palmitoyl-EA
3-ketosphinganine
Sphingosine
12-amino-octadecanoic acid
2-amino-octadecanoic acid
Sphingosine
5-hydroxy,3E-sphingosine
(8Z,d18:1) sphingosine

Experimental mass: 572.4878
Fold change (TA+N / TN): 0.47355
P value: 0.0085978
Arachidonic acid leelamide

Experimental mass: 591.4036
Fold change (TA+N / TN): 0.32054
P value: 0.010837
PA(12:0/16:1(9Z))
PA(13:0/15:1(9Z))
PA(14:0/14:1(9Z))
PA(14:1(9Z)/14:0)

PA(15:1(9Z)/13:0)
PA(16:1(9Z)/12:0)

Experimental mass: 605.4216
Fold change (TA+N / TN): 0.42609
P value: 0.01156
PA(17:1(9Z)/12:0)
PA(12:0/17:1(9Z))
PA(13:0/16:1(9Z))
PA(14:0/15:1(9Z))
PA(14:1(9Z)/15:0)
PA(15:0/14:1(9Z))
PA(15:1(9Z)/14:0)
PA(16:1(9Z)/13:0)

Experimental mass: 933.5437
Fold change (TA+N / TN): 0.49111
P value: 0.012819
PI(20:5(5Z,8Z,11Z,14Z,17Z)/22:4(7Z,10Z,13Z,16Z))
PI(22:4(7Z,10Z,13Z,16Z)/20:5(5Z,8Z,11Z,14Z,17Z))
PI(22:6(4Z,7Z,10Z,13Z,16Z,19Z)/20:3(8Z,11Z,14Z))
(Partial listing)

Experimental mass: 530.3286
Fold change (TA+N / TN): 0.49079
P value: 0.023337
LysoPE(0:0/22:4(7Z,10Z,13Z,16Z))
PE(22:4(7Z,10Z,13Z,16Z)/0:0)
Gymnodimine

Experimental mass: 644.4963
Fold change (TA+N / TN): 0.36271
P value: 0.026068
PE-NMe(O-14:0/O-14:0)

Experimental mass: 801.5914
Fold change (TA+N / TN): 0.4453
P value: 0.031291
PG(O-16:0/21:0)
PG(O-18:0/19:0)
PG(O-20:0/17:0)
all-trans decaprenyl phosphate
Decaprenyl phosphate

Experimental mass: 700.4934
Fold change (TA+N / TN): 0.48083
P value: 0.034981
PE(15:1(9Z)/18:2(9Z,12Z))
PC(18:3(6Z,9Z,12Z)/12:0)
PE(16:1(9Z)/17:2(9Z,12Z))
(Partial listing)

Experimental mass: 390.2368
Fold change (TA+N / TN): 0.48305
P value: 0.039787
C17 Sphinganine-1-phosphate

Experimental mass: 424.3039
Fold change (TA+N / TN): 0.31603
P value: 0.046954
thio-Miltefosine
3-hydroxypentadecanoyl carnitine

Negative mode

Experimental mass: 1063.7471
Fold change (TA+N / TN): 0.47802
P value: 0.0008945
TG(22:4(7Z,10Z,13Z,16Z)/22:5(7Z,10Z,13Z,16Z,19Z)/22:6(4Z,7Z,10Z,13Z,16Z,19Z))[iso6]
TG(22:5(7Z,10Z,13Z,16Z,19Z)/22:5(4Z,7Z,10Z,13Z,16Z)/22:5(7Z,10Z,13Z,16Z,19Z))
TG(22:5(7Z,10Z,13Z,16Z,19Z)/22:5(7Z,10Z,13Z,16Z,19Z)/22:5(7Z,10Z,13Z,16Z,19Z))
TG(22:5(4Z,7Z,10Z,13Z,16Z)/22:5(4Z,7Z,10Z,13Z,16Z)/22:5(4Z,7Z,10Z,13Z,16Z))
TG(22:5(4Z,7Z,10Z,13Z,16Z)/22:5(7Z,10Z,13Z,16Z,19Z)/22:5(4Z,7Z,10Z,13Z,16Z))
TG(22:5(4Z,7Z,10Z,13Z,16Z)/22:5(4Z,7Z,10Z,13Z,16Z)/22:5(7Z,10Z,13Z,16Z,19Z))
TG(22:5(4Z,7Z,10Z,13Z,16Z)/22:5(7Z,10Z,13Z,16Z,19Z)/22:5(7Z,10Z,13Z,16Z,19Z))
TG(22:4(7Z,10Z,13Z,16Z)/22:5(7Z,10Z,13Z,16Z,19Z)/22:6(4Z,7Z,10Z,13Z,16Z,19Z))
TG(22:4(7Z,10Z,13Z,16Z)/22:5(4Z,7Z,10Z,13Z,16Z)/22:6(4Z,7Z,10Z,13Z,16Z,19Z))
TG(22:4(7Z,10Z,13Z,16Z)/22:6(4Z,7Z,10Z,13Z,16Z,19Z)/22:5(7Z,10Z,13Z,16Z,19Z))
TG(22:5(4Z,7Z,10Z,13Z,16Z)/22:4(7Z,10Z,13Z,16Z)/22:6(4Z,7Z,10Z,13Z,16Z,19Z))
TG(22:5(7Z,10Z,13Z,16Z,19Z)/22:4(7Z,10Z,13Z,16Z)/22:6(4Z,7Z,10Z,13Z,16Z,19Z))
TG(22:4(7Z,10Z,13Z,16Z)/22:6(4Z,7Z,10Z,13Z,16Z,19Z)/22:5(4Z,7Z,10Z,13Z,16Z))

Experimental mass: 630.6242
Fold change (TA+N / TN): 0.44392
P value: 0.0010138
Cer(m18:1(4E)/24:1(15Z))

Experimental mass: 714.6509
Fold change (TA+N / TN): 0.49265
P value: 0.0011828
Cer(d18:0/26:0)
Cer(d20:0/24:0)

Experimental mass: 666.5904
Fold change (TA+N / TN): 0.38035
P value: 0.0013212
Cer(m18:1(4E)/24:1(15Z))

Experimental mass: 374.2438
Fold change (TA+N / TN): 0.47867
P value: 0.0013433
N-oleoyl glycine

Experimental mass: 712.6372
Fold change (TA+N / TN): 0.48793
P value: 0.0015323
Ceramide (d18:1/26:0)
Cer(d18:1/26:0)
Cer(d18:0/26:1(17Z))

Experimental mass: 672.6115
Fold change (TA+N / TN): 0.45032
P value: 0.0015373
Cer(d18:0/23:0)

Experimental mass: 1016.7482
Fold change (TA+N / TN): 0.49017
P value: 0.0015601
NAPE(18:1(9Z)/16:1(9Z)/18:0)

Experimental mass: 656.6285
Fold change (TA+N / TN): 0.47662
P value: 0.0018678
DG(19:0/0:0/19:0) (d5)

Experimental mass: 846.5826
Fold change (TA+N / TN): 0.49887
P value: 0.0018927
PE(21:0/20:3(8Z,11Z,14Z))
PC(18:2(9Z,12Z)/20:1(13Z))
PE(19:1(9Z)/22:2(13Z,16Z))
PE(22:2(13Z,16Z)/19:1(9Z))
PE(20:3(8Z,11Z,14Z)/21:0)

PC(18:0/20:3(5Z,11Z,14Z))
PC(18:0/20:3(5Z,8Z,14Z))
PC(20:3(8Z,11Z,14Z)/18:0)
PC(18:2(9Z,12Z)/20:1(11Z))
PC(18:1(11Z)/20:2(11Z,14Z))
PC(18:3(6Z,9Z,12Z)/20:0)
PC(20:2(11Z,14Z)/18:1(11Z))
PC(20:3(5Z,8Z,11Z)/18:0)
PC(18:3(9Z,12Z,15Z)/20:0)
PC(16:0/22:3(13Z,16Z,19Z))[U]
PC(18:0/20:3(5E,8E,11E))[U]
PC(18:0/20:3(5Z,8Z,11Z))[U]
PC(18:0/20:3(8Z,11Z,14Z))[U]
PC(16:1(9Z)/22:2(13Z,16Z))
PC(22:2(13Z,16Z)/16:1(9Z))
PC(20:0/18:3(6Z,9Z,12Z))
PC(20:0/18:3(9Z,12Z,15Z))
PC(20:1(11Z)/18:2(9Z,12Z))
PC(20:2(11Z,14Z)/18:1(9Z))
PC(18:0/20:3(5Z,8Z,11Z))
PC(18:0/20:3(8Z,11Z,14Z))
PC(18:1(9Z)/20:2(11Z,14Z))

Experimental mass: 724.5271
Fold change (TA+N / TN): 0.37514
P value: 0.0022509
PE(20:3(5Z,8Z,11Z)/P-16:0)
PE(P-18:1(11Z)/18:2(9Z,12Z))
PE(18:2(9Z,12Z)/P-18:1(11Z))
PE(P-18:1(9Z)/18:2(9Z,12Z))
PE(P-16:0/20:3(5Z,8Z,11Z))
PE(P-16:0/20:3(8Z,11Z,14Z))
PE(P-18:0/18:3(6Z,9Z,12Z))
PE(P-18:0/18:3(9Z,12Z,15Z))
PE(18:2(9Z,12Z)/P-18:1(9Z))
PE(20:3(8Z,11Z,14Z)/P-16:0)
PE(18:3(6Z,9Z,12Z)/P-18:0)
PE(18:3(9Z,12Z,15Z)/P-18:0)
PE(O-16:0/20:4(5Z,8Z,11Z,14Z))
PE(O-18:0/18:4(6Z,9Z,12Z,15Z))

Experimental mass: 730.5394
Fold change (TA+N / TN): 0.44441
P value: 0.0025185
PC(18:1(11Z)/14:0)
PC(14:1(9Z)/18:0)

PC(18:1(9Z)/14:0)
PC(14:0/18:1(9E))[U]
PC(14:0/18:1(9Z))[U]
PC(16:0/16:1(9Z))[U]
PC(18:1(9Z)/14:0)[U]
PC(14:0/18:1(11Z))
PC(14:0/18:1(9Z))
PE(20:1(11Z)/15:0)
PC(16:1(9Z)/16:0)
PE-NMe(16:0/18:1(9Z))
PC(16:0/16:1(9Z))
PC(18:0/14:1(9Z))
PE(15:0/20:1(11Z))
PE(15:1(9Z)/20:0)
PE(16:0/19:1(9Z))
PE(19:0/16:1(9Z))
PE(16:1(9Z)/19:0)
PC(15:0/17:1(9Z))
PE(22:1(11Z)/13:0)
PE(19:1(9Z)/16:0)
PC(15:1(9Z)/17:0)
PE(20:0/15:1(9Z))
PE(17:1(9Z)/18:0)
PE-NMe(18:1(9Z)/16:0)
PC(17:0/15:1(9Z))
PC(19:1(9Z)/13:0)
PC(19:1(9Z)/13:0)
PE(18:1(9Z)/17:0)
PC(17:1(9Z)/15:0)
PE(13:0/22:1(11Z))
PC(20:1(11Z)/12:0)
PE(17:0/18:1(9Z))
PC(12:0/20:1(11Z))
PE(14:1(9Z)/21:0)
PC(13:0/19:1(9Z))
PE(21:0/14:1(9Z))
PE-NMe(16:0/18:1(9Z))[U]
1,2-Dierucoyl-SN-Glycero-3-Phosphoethanolamine

Experimental mass: 1106.7628
Fold change (TA+N / TN): 0.49041
P value: 0.0028715
Trihexosylceramide (d18:1/22:0)
Manalpha1-3Manbeta1-4Glcbeta-Cer(d18:1/22:0)
Galalpha1-4Galbeta1-4Glcbeta-Cer(d18:1/22:0)
Galalpha1-3Galbeta1-4Glcbeta-Cer(d18:1/22:0)

Experimental mass: 726.5384
Fold change (TA+N / TN): 0.39339
P value: 0.0029261
PE(18:1(9Z)/P-18:1(11Z))
PE(20:2(11Z,14Z)/P-16:0)
PE(18:1(9Z)/P-18:1(9Z))
PE(18:1(11Z)/P-18:1(11Z))
PE(P-18:1(11Z)/18:1(11Z))
PE(18:1(11Z)/P-18:1(9Z))
PE(P-18:1(9Z)/18:1(9Z))
PE(18:2(9Z,12Z)/P-18:0)
PE(P-16:0/20:2(11Z,14Z))
PE(P-18:1(9Z)/18:1(11Z))
PE(P-18:0/18:2(9Z,12Z))
PE(P-18:1(11Z)/18:1(9Z))
PC(P-16:0/17:2(9Z,12Z))
PE(O-18:0/18:3(6Z,9Z,12Z))
PE(O-18:0/18:3(9Z,12Z,15Z))
PE(O-16:0/20:3(8Z,11Z,14Z))

Experimental mass: 977.7645
Fold change (TA+N / TN): 0.49521
P value: 0.0033399
TG(20:3n6/22:5(4Z,7Z,10Z,13Z,16Z)/20:4(5Z,8Z,11Z,14Z))
TG(20:2n6/22:6(4Z,7Z,10Z,13Z,16Z,19Z)/20:4(5Z,8Z,11Z,14Z))
TG(20:5(5Z,8Z,11Z,14Z,17Z)/20:5(5Z,8Z,11Z,14Z,17Z)/22:2(13Z,16Z))[iso3]
(Partial listing)

Experimental mass: 802.464
Fold change (TA+N / TN): 0.40117
P value: 0.0037708
PS(20:4(5Z,8Z,11Z,14Z)/18:4(6Z,9Z,12Z,15Z))
PS(18:3(6Z,9Z,12Z)/20:5(5Z,8Z,11Z,14Z,17Z))
PS(20:5(5Z,8Z,11Z,14Z,17Z)/18:3(6Z,9Z,12Z))
PS(20:5(5Z,8Z,11Z,14Z,17Z)/18:3(9Z,12Z,15Z))
PS(18:3(9Z,12Z,15Z)/20:5(5Z,8Z,11Z,14Z,17Z))
PS(18:4(6Z,9Z,12Z,15Z)/20:4(5Z,8Z,11Z,14Z))

Experimental mass: 1132.783
Fold change (TA+N / TN): 0.48535
P value: 0.0042804
Trihexosylceramide (d18:1/24:1(15Z))
Manalpha1-3Manbeta1-4Glcbeta-Cer(d18:1/24:1(15Z))
Galalpha1-4Galbeta1-4Glcbeta-Cer(d18:1/24:1(15Z))
Galalpha1-3Galbeta1-4Glcbeta-Cer(d18:1/24:1(15Z))

Experimental mass: 706.5428
Fold change (TA+N / TN): 0.45163
P value: 0.0043004
PS(O-16:0/O-16:0)[U]

Experimental mass: 744.5124
Fold change (TA+N / TN): 0.38721
P value: 0.0043082
PS(O-16:0/18:2(9Z,12Z))
PS(P-18:0/16:1(9Z))
PS(P-20:0/14:1(9Z))
PS(P-16:0/18:1(9Z))

Experimental mass: 978.7663
Fold change (TA+N / TN): 0.48458
P value: 0.0043119
PE(24:0/26:0)[U]
PE(25:0/25:0)[U]
PE(26:0/24:0)[U]
PC(21:0/26:0)[U]
PC(22:0/25:0)[U]
PC(23:0/24:0)[U]
PC(24:0/23:0)[U]
PC(25:0/22:0)[U]
PC(26:0/21:0)[U]

Experimental mass: 919.8131
Fold change (TA+N / TN): 0.49921
P value: 0.0043614
TG(22:5(7Z,10Z,13Z,16Z,19Z)/18:1(9Z)/o-18:0)
TG(18:2(9Z,12Z)/22:4(7Z,10Z,13Z,16Z)/o-18:0)
TG(18:2(9Z,12Z)/o-18:0/22:4(7Z,10Z,13Z,16Z))
(Partial listing)

Experimental mass: 690.5503
Fold change (TA+N / TN): 0.46887
P value: 0.0048147
PC(O-14:0/16:0)[U]
PC(O-14:0/16:0)
PE(O-18:0/15:0)
PE(O-20:0/13:0)
PE(O-16:0/17:0)
PC(O-16:0/14:0)
PC(O-18:0/12:0)

Experimental mass: 790.4361
Fold change (TA+N / TN): 0.398
P value: 0.0048494

PS(22:4(7Z,10Z,13Z,16Z)/12:0)
PS(12:0/22:4(7Z,10Z,13Z,16Z))
PS(18:3(6Z,9Z,12Z)/16:1(9Z))
PS(17:2(9Z,12Z)/17:2(9Z,12Z))
PS(16:0/18:4(6Z,9Z,12Z,15Z))
PS(18:4(6Z,9Z,12Z,15Z)/16:0)
PS(16:1(9Z)/18:3(6Z,9Z,12Z))
PS(20:3(8Z,11Z,14Z)/14:1(9Z))
PS(20:4(5Z,8Z,11Z,14Z)/14:0)
PS(14:1(9Z)/20:3(8Z,11Z,14Z))
PS(18:3(9Z,12Z,15Z)/16:1(9Z))
PS(14:0/20:4(5Z,8Z,11Z,14Z))
PS(16:1(9Z)/18:3(9Z,12Z,15Z))

Experimental mass: 829.4936
Fold change (TA+N / TN): 0.49223
P value: 0.005139

PI(18:3(9Z,12Z,15Z)/16:1(9Z))
PI(14:0/20:4(5Z,8Z,11Z,14Z))
PI(18:4(6Z,9Z,12Z,15Z)/16:0)
PI(16:1(9Z)/18:3(6Z,9Z,12Z))
PI(16:1(9Z)/18:3(9Z,12Z,15Z))
PI(22:4(7Z,10Z,13Z,16Z)/12:0)
PI(12:0/22:4(7Z,10Z,13Z,16Z))
PI(20:3(8Z,11Z,14Z)/14:1(9Z))
PI(20:4(5Z,8Z,11Z,14Z)/14:0)
PI(18:3(6Z,9Z,12Z)/16:1(9Z))
PI(17:2(9Z,12Z)/17:2(9Z,12Z))
PI(14:1(9Z)/20:3(8Z,11Z,14Z))
PI(16:0/18:4(6Z,9Z,12Z,15Z))
PGP(16:0/18:0)
PGP(18:0/16:0)
PI(O-16:0/16:1(9Z))
PI(O-18:0/14:1(9Z))
PI(P-16:0/16:0)
PI(P-18:0/14:0)
PI(P-20:0/12:0)
SQDG(16:0/16:0)
1,2-Di-O-palmitoyl-3-O-(6-sulfoquinovopyranosyl)glycerol

Experimental mass: 1036.734
Fold change (TA+N / TN): 0.49118
P value: 0.0051402
    FMC-6(d18:1/24:0(2-OH))
    LacCer(d18:1/26:0)
    LacCer(d18:0/26:1)
    Galalpha1-4Galbeta-Cer(d18:1/26:0)
    Manbeta1-4Glcbeta-Cer(d18:1/26:0)
    Galabiosylceramide (d18:1/26:0)
    Lactosylceramide (d18:1/26:0)

Experimental mass: 756.5168
Fold change (TA+N / TN): 0.48914
P value: 0.0052972
    PS(P-18:0/17:2(9Z,12Z))

Experimental mass: 662.5535
Fold change (TA+N / TN): 0.45082
P value: 0.0059028
    PE(O-16:0/O-16:0)[U]
    PE(O-16:0/O-16:0)
    Cer(t18:0/20:0(2OH))
    Cer(t20:0/18:0(2OH))

Experimental mass: 804.4608
Fold change (TA+N / TN): 0.43847
P value: 0.0060608
    PS(17:0/18:4(6Z,9Z,12Z,15Z))
    PS(17:1(9Z)/18:3(6Z,9Z,12Z))
    PS(17:1(9Z)/18:3(9Z,12Z,15Z))
    PS(22:4(7Z,10Z,13Z,16Z)/13:0)
    PS(17:2(9Z,12Z)/18:2(9Z,12Z))
    PS(20:3(8Z,11Z,14Z)/15:1(9Z))
    PS(13:0/22:4(7Z,10Z,13Z,16Z))
    PS(18:2(9Z,12Z)/17:2(9Z,12Z))
    PS(20:4(5Z,8Z,11Z,14Z)/15:0)
    PS(18:3(6Z,9Z,12Z)/17:1(9Z))
    PS(18:3(9Z,12Z,15Z)/17:1(9Z))
    PS(15:0/20:4(5Z,8Z,11Z,14Z))
    PS(18:4(6Z,9Z,12Z,15Z)/17:0)
    PS(15:1(9Z)/20:3(8Z,11Z,14Z))

Experimental mass: 778.4682
Fold change (TA+N / TN): 0.45468
P value: 0.0076298
    PS(22:6(4Z,7Z,10Z,13Z,16Z,19Z)/14:0)

PS(14:0/22:6(4Z,7Z,10Z,13Z,16Z,19Z))
PS(18:3(9Z,12Z,15Z)/18:3(9Z,12Z,15Z))
PS(18:2(9Z,12Z)/18:4(6Z,9Z,12Z,15Z))
PS(18:3(6Z,9Z,12Z)/18:3(6Z,9Z,12Z))
PS(18:3(6Z,9Z,12Z)/18:3(9Z,12Z,15Z))
PS(20:5(5Z,8Z,11Z,14Z,17Z)/16:1(9Z))
PS(18:3(9Z,12Z,15Z)/18:3(6Z,9Z,12Z))
PS(18:4(6Z,9Z,12Z,15Z)/18:2(9Z,12Z))
PS(16:1(9Z)/20:5(5Z,8Z,11Z,14Z,17Z))

Experimental mass: 780.5185
Fold change (TA+N / TN): 0.44246
P value: 0.007689
PE(18:0/20:4(5Z,8Z,11Z,13E)(15Ke))

Experimental mass: 1003.776
Fold change (TA+N / TN): 0.48768
P value: 0.0079988
TG(22:4(7Z,10Z,13Z,16Z)/20:4(8Z,11Z,14Z,17Z)/22:5(7Z,10Z,13Z,16Z,19Z))
TG(22:5(4Z,7Z,10Z,13Z,16Z)/20:3(5Z,8Z,11Z)/22:5(7Z,10Z,13Z,16Z,19Z))
TG(20:3n6/22:4(7Z,10Z,13Z,16Z)/22:6(4Z,7Z,10Z,13Z,16Z,19Z))
(Partial listing)

Experimental mass: 734.5017
Fold change (TA+N / TN): 0.46628
P value: 0.0080514
PS(16:0/16:0)
PS(16:0/16:0)
PS(14:0/18:0)
PS(18:0/14:0)
PS(16:0/16:0)
PS(20:0/12:0)
PS(19:0/13:0)
PS(17:0/15:0)
PS(15:0/17:0)
PS(13:0/19:0)
PS(12:0/20:0)

Experimental mass: 782.5082
Fold change (TA+N / TN): 0.41898
P value: 0.0093905
PS(O-20:0/14:1(9Z))
PS(O-18:0/16:1(9Z))
PS(O-16:0/18:1(9Z))
PS(P-16:0/18:0)
PS(P-18:0/16:0)

PS(P-20:0/14:0)

Experimental mass: 752.4673
Fold change (TA+N / TN): 0.46172
P value: 0.0097428
PS(P-16:0/16:1(9Z))
PS(P-18:0/14:1(9Z))

Experimental mass: 604.6007
Fold change (TA+N / TN): 0.47274
P value: 0.010536
Cer(m18:1(4E)/22:0)

Experimental mass: 810.4087
Fold change (TA+N / TN): 0.43473
P value: 0.013276
PS(18:4(6Z,9Z,12Z,15Z)/18:4(6Z,9Z,12Z,15Z))

Experimental mass: 836.4227
Fold change (TA+N / TN): 0.44307
P value: 0.015383
PS(20:5(5Z,8Z,11Z,14Z,17Z)/18:4(6Z,9Z,12Z,15Z))
PS(18:4(6Z,9Z,12Z,15Z)/20:5(5Z,8Z,11Z,14Z,17Z))

Experimental mass: 676.5301
Fold change (TA+N / TN): 0.4949
P value: 0.016973
PE(O-16:0/16:0)[U]
PE(O-18:0/14:0)
PE(O-20:0/12:0)
PE(O-16:0/16:0)
PC(O-14:0/15:0)
PC(O-16:0/13:0)

Experimental mass: 736.4969
Fold change (TA+N / TN): 0.46899
P value: 0.023788
PE(22:5(7Z,10Z,13Z,16Z,19Z)/14:0)
PE(18:1(11Z)/18:4(6Z,9Z,12Z,15Z))
PE(18:4(6Z,9Z,12Z,15Z)/18:1(9Z))
PE(22:4(7Z,10Z,13Z,16Z)/14:1(9Z))
PE(16:1(9Z)/20:4(8Z,11Z,14Z,17Z))
PE(14:0/22:5(7Z,10Z,13Z,16Z,19Z))
PE(22:5(4Z,7Z,10Z,13Z,16Z)/14:0)
PE(20:4(8Z,11Z,14Z,17Z)/16:1(9Z))
PE(18:1(9Z)/18:4(6Z,9Z,12Z,15Z))

PE(18:4(6Z,9Z,12Z,15Z)/18:1(11Z))
PE(18:2(9Z,12Z)/18:3(6Z,9Z,12Z))
PE(18:2(9Z,12Z)/18:3(9Z,12Z,15Z))
PE(14:0/22:5(4Z,7Z,10Z,13Z,16Z))
PE(20:4(5Z,8Z,11Z,14Z)/16:1(9Z))
PE(18:3(6Z,9Z,12Z)/18:2(9Z,12Z))
PE(16:1(9Z)/20:4(5Z,8Z,11Z,14Z))
PE(14:1(9Z)/22:4(7Z,10Z,13Z,16Z))
PE(16:0/20:5(5Z,8Z,11Z,14Z,17Z))
PE(20:5(5Z,8Z,11Z,14Z,17Z)/16:0)
PE(18:3(9Z,12Z,15Z)/18:2(9Z,12Z))
PC(20:5(5Z,8Z,11Z,14Z,17Z)/13:0)
PC(15:1(9Z)/18:4(6Z,9Z,12Z,15Z))
PC(18:4(6Z,9Z,12Z,15Z)/15:1(9Z))
PC(13:0/20:5(5Z,8Z,11Z,14Z,17Z))

Supplemental File S2: List of masses identified to be differentially affected by TN vs. TA+N that correspond to peptides

Positive mode

Experimental mass: 293.1363
Fold change (TA+N / TN): 0.48969
P value: 0.00021073
- Histidinyl-Histidine
- Coriandrone B
- 11,12-Dimethoxydihydrokawain
- Coriandrone A
- Tetrahydrotrimethylhispidin

Negative mode

Experimental mass: 790.4361
Fold change (TA+N / TN): 0.398
P value: 0.0048494
- DHOAA(AcO)-Val-Phe(NMe)-Pro-Phe(NMe)-Gly-OMe

Experimental mass: 267.1452
Fold change (TA+N / TN): 0.48336
P value: 0.0074803
- Histidylleucine;
- Histidinyl-Leucine
- Leucyl-Histidine
- Histidinyl-Isoleucine

Experimental mass: 1200.3263
Fold change (TA+N / TN): 0.49912
P value: 0.013454
- UDPMurNAc(oyl-L-Ala-gamma-D-Glu-L-Lys-D-Ala-D-Ser)
